# Regional Economic Prediction Model Using Backpropagation Integrated with Bayesian Vector Neural Network in Big Data Analytics

**DOI:** 10.1155/2022/1438648

**Published:** 2022-02-16

**Authors:** Qisong Zhang, Lei Yan, Runbo Hu, Yingqiu Li, Li Hou

**Affiliations:** ^1^School of Information and Business Management, Dalian Neusoft University of Information, Dalian 116000, Liaoning, China; ^2^Business School, Zhejiang Wanli University, Ningbo 315000, Zhejiang, China; ^3^China Business Executives Academy, Dalian 116000, Liaoning, China; ^4^School of Economics and Management, Ningbo University of Technology, Ningbo 315000, Zhejiang, China

## Abstract

Forecasting economic growth is critical for formulating national economic development policies. Neural Networks are a type of artificial intelligence that may be used to model complex target functions. ANN (Artificial Neural Networks) are one of the most effective learning approaches now available for specific sorts of tasks, such as learning to understand complex real-world sensor data. This paper proposes the regional economic prediction model based on neural networks techniques. Bayesian vector neural network (BVNN) is integrated with backpropagation (BP) model. The database has been collected based on the economics of particular region which has been extracted and classified using knowledge-based computer analysis by neural networks. Discretization, reduction, importance ranking, and prediction rule are attributes considered here. Then, as the input training sample, feed extracted important components into the NN. This strategy enhanced the training speed and prediction accuracy by reducing structure of NN. WEO, APDREO, and AFRREO are the dataset and FWA-SVR and LSTM are the existing method taken for comparison. For the WEO dataset, 97% of GDP and 98% of accuracy are produced. For APDREO dataset, 92% of accuracy and GDP of 97% are obtained. For AFRREO dataset, 98% of accuracy is produced. The neural network can tackle nonlinear problems, according to experimental data, and the technology has been proven to be successful and viable with high accuracy. For practical application, the model has a good reference value. The proposed model reduces error by increasing the convergence rate and accuracy for each dataset.

## 1. Introduction

In evolution of human society, economic gain is a critical topic [1]. Based on surroundings, economic system is constantly influenced, which is also a nonlinear system. Additional interference variables, as well as different reasons that lead to ambiguous and inadequate historical information required for macroeconomic modelling [2], act directly on entire development of macroeconomic scheme process. As a result, the traditional BP_NN regional economic trend prediction technique cannot effectively correct weight in analysis process of vast data and operation efficiency is low. A typical BP_NN learning process includes two stages: forward data propagation and backward error propagation. Input signal is analysed by neurons in input and hidden layers before being sent to output layer to display result [3]. It uses reverse propagation procedure if intended output is not accessible at output layer. Along originally linked path, there is a difference between network output and returned actual value. Modifying weights of neurons at each layer can lessen inaccuracy. Return to forward propagation process and continue until error is less than specified value. As a result, an enhanced BP_NN based approach for predicting regional economic trend is created, providing a solid foundation for regional economic development [4]. Forecasting regional economic activity is an increasingly significant component of regional economic research. Regional economic predictions can directly aid local, subnational, and national policy makers as well as concerned business executives. Both policymakers and business executives need accurate predictions of key economic aggregates, such as employment, income, and output for medium-long term planning purposes. Regional economic activity forecasts have been used to explain macroeconomic forces, including predicting the stock market and cyclicality of national labour market movements. Furthermore, international investors and multinational agencies engaged in megaprojects at a regional level also need accurate predictions for investment planning reasons [5].

There are numerous statistical tools and models available today for assessing and developing integral indicators of quality of life. Most methods, however, have limitations in terms of applicability and do not function with incomplete or “noisy” data. As a result, neural network technologies and intelligent information systems based on them are presented as an alternate strategy for handling semistructured and normalised challenges of analysing quality of life and developing forecasting models [6–8]. Savings, housing, socioeconomic features of the population, the dynamics, and differentiation of these indicators in the context of demographic groupings and regional economies are the subjects of several studies [9]. Systems of absolute and relative indices are established in these studies to characterize the degree of provision and fulfilment of the demands of various groups of people. Authors use ways to identify the concepts of “cost of living” and “quality of life” in several models of the standard of living.

The cost of living is defined in classical economics as the value of a set of commodities that is determined, first and foremost, by the level of food and service costs, as well as changes in types of income, consumption habits, tax legislation, and demographic indicators of population groupings. Consumer basket and commodity bundle, as well as living wage [10], are the major estimated rates. The presence of structural shifts and the interaction between different sets of indicators, as well as nonlinear dependencies between indicators, are characteristics of time series of socioeconomic indicators [11–13]. Therefore, linear models are not always able to give qualitative results. In this regard, a hybrid approach based on the construction of an ensemble of hybrid models is promising, in which, along with regression models, models based on neural networks, decision trees, and neurofuzzy networks are used. In recent years, in many works, the hybrid approach has been developed to forecast time series [14–17]. Statistics mining, connection mining, decision tree mining, NNM, and other techniques are used in data mining. ANN is an auto adapted dynamics system, which is one of these approaches. ANNs have developed as a potent statistical modelling technique and a highly powerful instrument in modern quantitative economics.

Various economic forecasting methods are presented to anticipate future growth of related industries based on existing state of industrial economy in terms of research methodologies. To forecast the tourism business, an FWA-SVR forecasting model was suggested. Grey model GM (1, 1) was used by [18] to forecast the growth tendencies of China's real estate economy. Another study looked at China's macroeconomic scheme by using a BMF-VAR method to forecast the country's macroeconomy [19]. To predict and analyse China inflation rate, deep learning based LSTM method was presented in [20]. The author of [21] built a MIDAS model for assessing and forecasting CPI and PPI using commodity price big data. The Chinese consumer confidence index was analysed and forecasted using a DNN_CNN-LSTM model in [22]. Paper [23] suggested an autoregressive (AR) model for more accurate forecasting of energy demand and supply. Paper [24] created a dataset of 77 countries covering over 90% of world GDP and used cross-country statistics to anticipate US economic growth during downturns. In [25], the author offered a machine learning technique for constructing mood indicators as well as economic forecasting using evolving confidence indicators. Paper [26] developed a self-correcting intelligent pandemic prediction model to improve COVID-19 forecast accuracy and strengthen economic management and control. Paper [27] used WEKA3.9 to create three prediction models to accurately predict business failure.

The FWA-SVM model has the smallest errors in predicting the GDP ratio and filling rate [28] while it keeps the shortest time to optimize the SVM parameters. The bandwidth requirement of LSTM [29] is very high and the computational accuracy is low in these systems in big data analytics so BVNN_BP is proposed to overcome these limitations.

The proposed contribution of this paper is as follows:

- To collect the database and process it with proposed Bayesian vector neural network (BVNN) and to integrate with backpropagation (BP) model by feature extraction and classification
- To compare the proposed BVNN_BP model with the existing system like FWA-SVR and LSTM with various datasets like WEO, APDREO, and AFRREO

However, understanding and applying these strategies necessitates a thorough understanding of mathematics, algorithm knowledge, and computer programme technology. Methodologies in the study case produce one type of simple and high-accuracy economic growth forecast.

## 2. Prediction Model Using Proposed Design of Bayesian Vector Neural Network (BVNN) with Backpropagation (BP)

### 2.1. Model Structure

Our BVNN_BP inputs are GDP growth index, primary, secondary, and tertiary industry growth value for *n* consecutive years in a region as panel data into the network to predict the output primary, and secondary and tertiary industry growth values for the region in year *n* + 1 with attribute discretization, reduction importance ranking, and prediction rule.

The model divides input data into two parts.

1. Regional GDP network: regional GDP data are independent of other indicator data before fully connected layer of the neural network, and feature information is extracted using BP, batch normalisation, and ReLU activation function. This design reflects that the regional GDP can mirror the overall economic level of the region, which is equivalent to gauging the regional economic development level by regional GDP to divide regions and using different forecasting strategies for regions with different development levels in the fully connected layer.
2. Regional panel data network: using neural network convolution kernels, the regional GDP growth index, primary, secondary, and tertiary industry growth values are integrated to produce panel data to detect industry correlations and effect of industry data on later growth values within *n* successive years. To extract time series data features as well as probable industry links, three BVNN layers are utilised first. (1) and (2) outputs values are inputs of fully connected layers and to enhance two networks multichannel outputs, GDP outputs are combined. The mathematical representation of BVNN layers is represented in Table1 with its algorithm.

### 2.2. Proposed Model Analysis

Let us consider each feature by a variable *X*_*i*_⊆*RR*. Then each client is totally defined by an n-dimensional vector. *x*=(*x*_1_ …, *x*_*n*_) ∈ *X*⊆*R*^*n*^.

Basic rule for calculating a unit's activation value in relation to other connected units with a strength *w*_*ji*_ is a function of weighted sum of inputs expressed [30] in

(1) $$\begin{array}{c} u_{j}=f\left(\sum_{i}u_{i}\cdot w_{ji}\right)=f\left({\text{Net}}_{j}\right)=\frac{1}{1+e^{-{\text{Net}}_{j}}}. \end{array}$$

Net_*j*_ = Net Input to *j* level unit.

To this equation, one must include the unit's threshold, which is defined as the unit's proclivity to activate or inhibit itself by

(2) $$\begin{array}{c} u_{j}=f\left({\text{Net}}_{j}\right)=\frac{1}{1+e}-\left(\sum \left(i\right)w_{ji}\cdot u_{i}+\theta_{j}\right). \end{array}$$

If ∂*u*_*j*_/∂Net_*j*_=*u*_*j*_ · (1 − *u*_*j*_) then Δout_*j*_ from (3) and (4):

(3) $$\begin{array}{c} \Delta out_{j}=\left(t_{j}-u_{j}\right)\cdot u_{j}\cdot \left(1-u_{j}\right),\\ \frac{\Delta w_{ji}}{\partial }=-\frac{\partial E_{p}}{\partial w_{ji}}. \end{array}$$

(4) $$\begin{array}{c} E_{p}=\frac{1}{2}\sum_{k}{\left(t_{pk}-u_{pk}\right)}^{2}\\ =\frac{1}{2}\sum_{k}\left(t_{pk}-f_{k}\left(1-u_{j}\right)\right)\\ =\frac{1}{2}\sum_{k}\left(t_{pk}-f_{k}\left(\sum_{i}w_{kj}\cdot u_{pj}+\theta_{k}\right)\right). \end{array}$$

*E* = Error; *p* = Model; *t*_*k*_ = Target; *u*_*k*_ = Output and then

(5) $$\begin{array}{c} \frac{\partial \left({\text{Net}}_{pk}\right)}{\partial w_{kj}}=\left(\frac{\partial }{\partial w_{kj}}\cdot \sum_{j}w_{kj}\cdot u_{pj}+\theta_{k}\right)\\ =u_{pj}=-\frac{\partial E_{p}}{\partial w_{kj}}\left(t_{pk}-u_{pk}\right)\cdot f_{k}^{\prime }\left({\text{Net}}_{pk}\right)\cdot u_{pj}. \end{array}$$

Value out_*j*_ will now determine the amount of value to be added or subtracted from weight *w*_*ji*_ in connection to activation state of unit *u*_*i*_, which is activation with which *u*_*j*_ is linked to weight *w*_*ji*_ and coefficient *r*. This is correction rate that should be used as shown in

(6) $$\begin{array}{c} \Delta w_{ji}=r\cdot \Delta {\text{out}}_{j}\cdot u_{j}. \end{array}$$

Δ*w*_*ji*_ can have a negative or positive value. It denotes the “quantum” that will be added or subtracted from the prior weight *w*_*ji*_ value by

(7) $$\begin{array}{c} w_{ji\left(n+1\right)}=w_{ji\left(n\right)}+\Delta w_{ji}. \end{array}$$

However, this assumption is only true for weights which connect a unit layer to output units layer. Correction approach outlined so far is only applied to BP with two layers, to use gradient descent [31] in (8) and (9).

(8) $$\begin{array}{c} \frac{\partial J}{\partial w_{11}^{1}}=\left(\frac{\partial J}{\partial a_{1}^{2}}\right)\left(\frac{\partial a_{i}^{2}}{\partial z}\right)\left(\frac{\partial z}{\partial w_{1}^{1}}\right)=\left(\frac{\partial \left(\left(1/2\right){\left(a_{1}^{2}-y\right)}^{2}\right)}{\partial a_{1}^{2}}\right)\left(\frac{\partial \left(g\left(z\right)\right)}{\partial z}\right)\left(\frac{\partial \left(a_{1}^{1}w_{11}^{1}+b^{1}\right)}{\partial w_{11}^{1}}\right)\\ =\left(\left(a_{1}^{2}-y\right)\right)\left(g\left(z\right)\left(1-g\left(z\right)\right)\right)\left(a_{1}^{1}\right)\\ =\left(g\left(z\right)-y\right)\cdot g\left(z\right)\left(1-g\left(z\right)\right)\cdot a_{1}^{1}. \end{array}$$

It can be easily shown using calculus that derivative of the sigmoid function *g*(*z*) is given by *g*(*z*). (1 − *g*(*z*)). Partial derivative of *J* is with respect to bias.

(9) $$\begin{array}{c} \frac{\partial J}{\partial b^{1}}=\left(\frac{\partial J}{\partial a_{1}^{2}}\right)\left(\frac{\partial a_{1}^{2}}{\partial z}\right)\left(\frac{\partial z}{\partial b^{1}}\right)\\ =\left(\frac{\partial \left(\left(1/2\right){\left(a_{1}^{2}-y\right)}^{2}\right)}{\partial a_{1}^{2}}\right)\left(\frac{\partial \left(g\left(z\right)\right)}{\partial z}\right)\left(\frac{\partial \left(a_{1}^{1}w_{1}^{1}+b^{1}\right)}{\partial b^{1}}\right)\\ =\left(\left(a_{1}^{2}-y\right)\right)\left(g\left(z\right)\left(1-g\left(z\right)\right)\right)\\ =\left(g\left(z\right)-y\right)\cdot g\left(z\right)\left(1-g\left(z\right)\right). \end{array}$$

In this network, (10) can be rewritten as

(10) $$\begin{array}{c} \frac{\partial J}{\partial w_{11}^{2}}=\left(\frac{\partial J}{\partial a_{1}^{2}}\right)\left(\frac{\partial a_{i}^{2}}{\partial z}\right)\left(\frac{\partial z}{\partial w_{11}}\right)\\ =\left(\frac{\partial \left(\left(1/2\right){\left(a_{1}^{3}-y\right)}^{2}\right)}{\partial a_{1}^{3}}\right)\left(\frac{\partial \left(g\left(z^{2}\right)\right)}{\partial z^{2}}\right)\left(\frac{\partial \left(a_{1}^{2}w_{11}^{2}+b^{2}\right)}{\partial w_{11}^{2}}\right)\\ =\left(\left(a_{1}^{3}-y\right)\right)\left(g\left(z^{2}\right)\left(1-g\left(z^{2}\right)\right)\right)\left(a_{1}^{2}\right)\\ =\left(g\left(z^{2}\right)-y\right)\cdot g\left(z^{2}\right)\left(1-g\left(z^{2}\right)\right)\cdot a_{1}^{2} \end{array}$$

So, calculating the required gradients can be done. This explains the basic mathematics behind backpropagation. Now, after understanding the above mathematical aspects, we can backpropagate a simple linear NN using this approach. For more complex NN, with multiple nodes in every layer, the approach remains the same, and the only difference we get is the changing of subscripts and superscripts of *w*, *z*, *a*, and *b*. This makes backpropagation complex, and we need to use matrix forms of the derivatives, i.e., gradient vectors. But the basic idea remains the same. Backpropagation is simply using the chain rule of derivatives to calculate the change of cost function *J* with respect to changing parameters of the network. The proposed BP algorithm helps to compute the gradient which can be used to tweak the parameters using gradient as shown in Algorithm 1. Difference between actual output and desired one and summation report are constructed using previously calculated error coefficient Δout_*j*_ and weight associated with that coefficient was referring [32] to

(11) $$\begin{array}{c} \Delta {\text{hidden}}_{i}=u_{i}\cdot \left(1-u_{i}\right)\cdot \sum \Delta {\text{out}}_{j}\cdot w_{ji}. \end{array}$$

And therefore,

(12) $$\begin{array}{c} \Delta w_{ik}=r\cdot \Delta {\text{hidden}}_{i}\cdot u_{k}. \end{array}$$

In fact, start from

(13) $$\begin{array}{c} \frac{\partial E_{p}}{\partial w_{kj}}=\frac{1}{2}\sum_{k}\frac{\partial }{\partial w_{kj}}\cdot \left(t_{pk}-u_{pk}\right)\\ =-\sum_{k}\left(t_{pk}-u_{pk}\right)\cdot f_{k}\left(Net_{pk}\right)\cdot w_{kj}\cdot f_{j}\left(Net_{pj}\right)\cdot u_{pi}. \end{array}$$

*w*_*kj*_ = Hidden-Outputs weights.

Weights associated with output are corrected in the calculation as shown in

(14) $$\begin{array}{c} \Delta w_{ji}=r\cdot \Delta {\text{out}}_{j}\cdot u_{i}. \end{array}$$

Weights that are not connected to output are corrected by

(15) $$\begin{array}{c} \Delta {\text{hidden}}_{i}=f^{\prime }\left(u_{i}\right)\cdot \sum_{j}\Delta {\text{out}}_{j}\cdot w_{ji}. \end{array}$$

Fulfilment of the corrections on weights is done by

(16) $$\begin{array}{c} w_{ij\left(n+1\right)}=w_{ij\left(n\right)}+\Delta {\text{out}}_{i}\cdot u_{j}\cdot \text{Rate}. \end{array}$$

For output units as shown

(17) $$\begin{array}{c} {\text{Bias}}_{j\left(n+1\right)}={\text{Bias}}_{j\left(n\right)}+\Delta {\text{hidden}}_{j}\cdot \text{Rate}. \end{array}$$

*x*_*t*,*i*_^*sum*^(*θ*) ∈ *R*^*n*^ for all *t*=1,2,…, *T*, where *T* is simulation length; *i* denotes random number initialized. In general, estimate or calibration processes try to find suitable values for *h* so that *X*^*sim*^(*θ*, *T*, *i*) creates dynamics that are as near to those observed in an experimentally measured counterpart as possible as shown in

(18) $$\begin{array}{c} X=\left[x_{1},x_{2},\ldots ,x_{T}\right]. \end{array}$$

*x*_*t*_ ∈ *R*^*n*^ for all *t*=1,2,…, *T*.

As a result, Bayesian evaluation is phrased in terms of Bayes' theorem as given in

(19) $$\begin{array}{c} p\left(\theta |X\right)=\frac{p\left(X|\theta \right)p\left(\theta \right)}{p\left(X\right)}. \end{array}$$

Method for approximating *p*(*θ|X*) for a certain value of *h* then allows us to calculate right-hand side of

(20) $$\begin{array}{c} p\left(\theta |X\right)\propto p\left(\theta |X\right)p\left(\theta \right). \end{array}$$

For all $t\geq \tilde{T}$ in which *x*_*t*,*i*_^*sim*^(*θ*) varies for stationary level, *E*[*x*_*t*,*i*_^*sim*^(*θ*)*|t* ≥ *T*] which allows us to further consider that $x_{\tilde{T},i}^{sim}\left(\theta \right),x_{\dot{T}+1}^{\sin}\left(\theta \right),\ldots ,x_{T,i}^{sim}\left(\theta \right)$ establishes a random sample from an assumed distribution. Using kernel density estimation (KDE), we can then establish a density function which designates this distribution, which we designate by $\tilde{f}\left(x|\theta \right)$ permitting us to estimated likelihood of empirically sampled data for a specified value of *h* as given in

(21) $$\begin{array}{c} p\left(X|\theta \right)=\prod_{t=1}^{T}\tilde{f}\left(x_{t}|\theta \right). \end{array}$$

For all *L* < *t* ≤ *T*, this means that *x*_*t*,*i*_^*sim*^(*θ*) is solely dependent on past *L* realised values. As a result, our objective is to evaluate above conditional densities by

(22) $$\begin{array}{c} \tilde{f}\left(x_{t-L,i}^{sim},\ldots ,x_{t-1,i}^{sim},x_{t,i}^{sim},\phi \right)≃p\left(x_{t,i}^{sim}|x_{t-L,i}^{sim},\ldots ,x_{t-1,i}^{sim}:\theta \right). \end{array}$$

For all L < t ≤ T, *ϕ*=*ϕ*(*θ*).

The aforementioned technique is made up of two main components: a mixture of K Gaussian random variables in Algorithm 2 and a random number generator as shown in

(23) $$\begin{array}{c} p\left(X|\theta \right)=\prod_{t=1}^{T-L}\tilde{f}\left(x_{t},\ldots ,x_{i+L-1},x_{i+L},\phi \right). \end{array}$$

This leads us to define following loss function as shown in

(24) $$\begin{array}{c} LS\left(\theta^{rrue\;},\hat{\theta }\right)=\theta^{true\;}-{\hat{\theta }}_{2}. \end{array}$$

## 3. Simulation Results

### 3.1. Dataset Description

#### 3.1.1. World Economic Outlook (WEO)

The WEO database is developed during the biennial WEO exercise, which takes place between January and June each year and culminates in the release of the WEO in April and September/October. A database format is provided for selected series from the publication.

#### 3.1.2. Asia and Pacific Regional Economic Outlook (APDREO)

APDREO offers data on recent economic trends and forecasts for Asian and Pacific countries. The data for REO for Asia and the Pacific is compiled in tandem with semiannual WEO exercises, which take place in the spring and fall. The data is in line with the WEO's projections. Due to variances in group membership, REO aggregate data may differ from WEO aggregate data. Weighted averages of data for specific countries make up composite data for country groups. All concepts are represented by arithmetic weighted averages, with the exception of inflation and broad money, which are represented by geometric averages.

#### 3.1.3. Sub-Saharan Africa Regional Economic Outlook (AFRREO)

AFRREO provides data on recent economic events and scenarios in Sub-Saharan African countries. The data for REO for Sub-Saharan Africa is compiled in tandem with semiannual WEO exercises, which take place in the spring and fall.

### 3.2. Comparative Analysis

This section discusses the comparative analysis for various datasets in terms of GDP per capita prediction, prediction accuracy, GDP per primary growth, GDP per secondary growth, and convergence rate. The techniques compared here are FWA-SVR and LSTM for WEO dataset, APDREO dataset, and AFRREO dataset. Python is the software used for implementation and the comparison results are obtained from the simulation.  Table 2 represents the comparative analysis of regional economic prediction model based parameters for WEO dataset.

**Figure 1:**
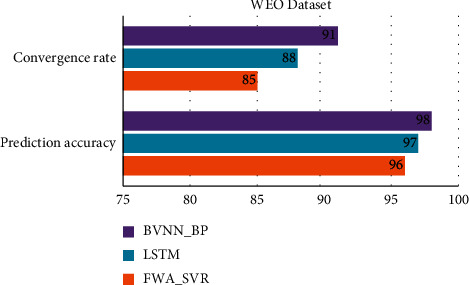
Comparative analysis of WEO dataset for convergence rate and prediction accuracy.

**Figure 2:**
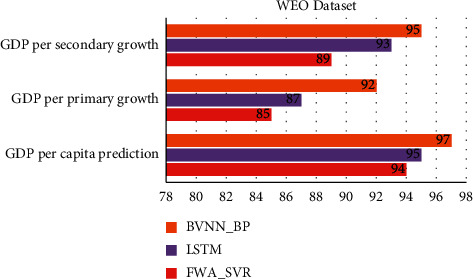
Comparative analysis of WEO dataset for regional economic prediction model.

Table 2 and Figures 1 and 2 show comparative analysis for proposed BVNN_BP with existing technique LSTM and FWA-SVR. The parameters are compared for WEO dataset with existing techniques. Based on this comparison, the proposed technique obtained optimal results in terms of predicting the economic rate of the region and in terms of accuracy for this prediction higher for proposed technique when compared with existing technique. GDP predicted per capita by BVNN_BP is 97%, and accuracy is 98% which is higher based on the existing technique comparison. In terms of primary and secondary growth of GDP, it is comparatively higher for proposed model. Convergence is also optimized by BVNN_BP up to 91%.  Table 1 shows the comparative analysis of regional economic prediction model based parameters for APDREO dataset.

**Figure 3:**
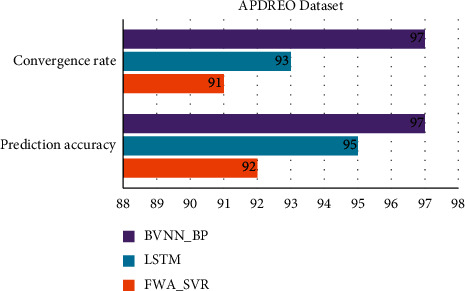
Comparative analysis of APDREO dataset for convergence rate and prediction accuracy.

**Figure 4:**
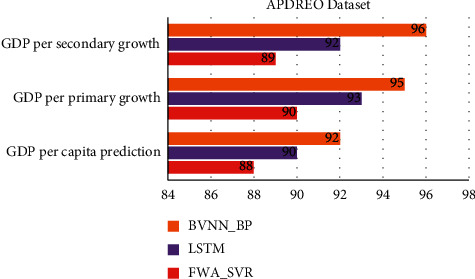
Comparative analysis of APDREO dataset for regional economic prediction model.

Table 1 and Figures 3 and 4 show comparative analysis for proposed BVNN_BP with existing technique LSTM and FWA-SVR for APDREO dataset. This dataset gives the economic analysis and data from Asia and Pacific region.

For this regional analysis the GDP prediction obtained by proposed BVNN_BP is 92%, accuracy predicted by this proposed technique is 97%, and based on primary and secondary growth of GDP for this region of 95% and 96%, the convergence rate here is 97%.

Table 3 represents the comparative analysis of regional economic prediction model based parameters for AFRREO dataset.

**Figure 5:**
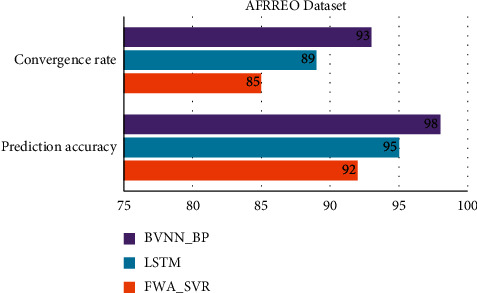
Comparative analysis of APDREO dataset for convergence rate and prediction accuracy.

**Figure 6:**
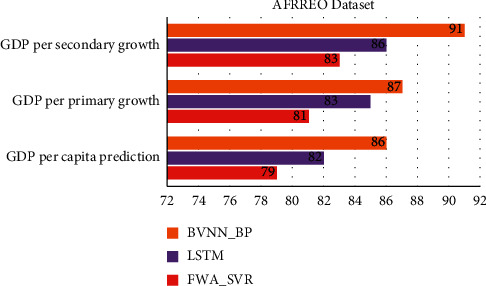
Comparative analysis of AFRREO dataset for regional economic prediction model.

Table 3 and Figures 5 and 6 show comparative analysis for proposed technique and existing technique for AFRREO dataset. This dataset covers the economic growth for Sub-Saharan Africa region. Economically Africa is considered to be poor country, so the GDP prediction based on both primary and secondary industry growth is essential for this region. By analysing the data of this region, the GDP prediction is optimal but not up to the mark to region analysis. The accuracy for this dataset by proposed model would be 98%.

## 4. Discussion

Country econometric models continue to play an important role in planning and forecasting key indicators of socioeconomic development. They allow taking into account the interdependencies of indicators associated with their economic content. At the same time, many economic time series are characterized by nonlinear dependencies, and the influencing factors are often impossible to describe explicitly in the form of regression equations. This makes, as shown by numerous studies, the application of neural network methods and technologies very promising.

Over the past decade, approaches to forecasting time series based on feedforward networks and recurrent networks have been developing, including their application for forecasting economic indicators. At the same time, machine learning platforms and tools are becoming widespread, making it possible to implement all the main architectures and methods of training neural networks. It should be noted that the use of neural network tools for separate unrelated time series does not allow for a systematic approach to modelling the economic sphere. We are convinced that a hybrid methodology should be applied with the possibility of choosing in each case the most appropriate method. Such an approach is implemented in developed Bayesian vector neural network (BVNN) which is integrated with backpropagation (BP) based feature extraction and classification. The suggested technique can forecast regional economy and increase prediction model's convergence rate without impacting accuracy, effectively enhance effectiveness of regional economic analysis and prediction, lower percentage error, and have advantages of greater operating effectiveness. The only limitation of the proposed model is that the convergence rate and accuracy are dependent on each other so separate works are needed to improve the accuracy and convergence rate.

## 5. Conclusion

This paper proposed novel technique regional economic prediction based on machine learning techniques. Data were first normalised to balance deviations among indicators and then normalised data were transferred exponentially to balance deviations among regions, with the goal of reducing data heterogeneity among economic indicators of each region and differences in economic development levels of each region. Database has been collected and processed with proposed Bayesian vector neural network (BVNN) which is integrated with backpropagation (BP) model by feature extraction and classification. The experimental results show the comparative analysis for various datasets in terms of GDP per capita prediction, prediction accuracy, GDP per primary growth, GDP per secondary growth, and convergence rate. The techniques compared here are FWA-SVR and LSTM for WEO dataset, APDREO dataset, and AFRREO dataset. The proposed model reduces error by increasing the convergence rate and accuracy for each dataset.

## Figures and Tables

**Algorithm 1:**
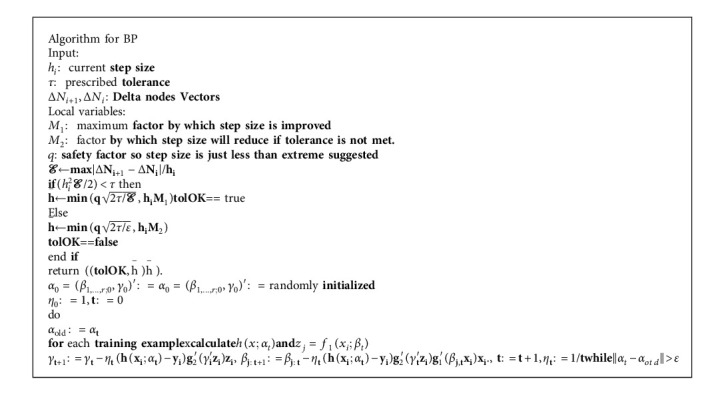
Proposed BP Algorithm.

**Algorithm 2:**
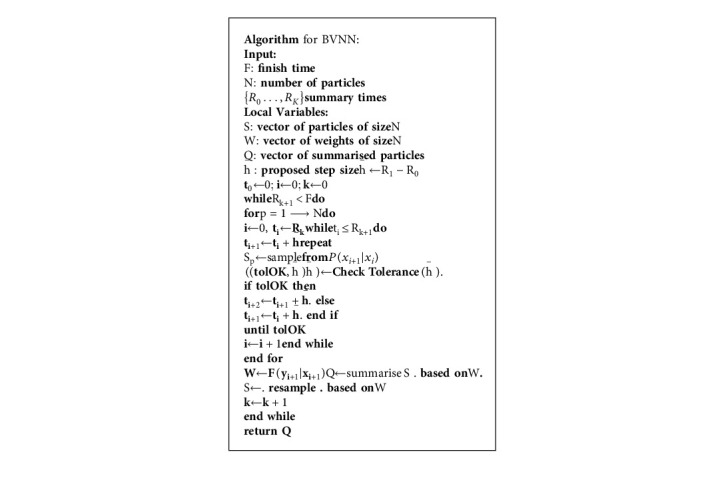
Proposed BVNN Algorithm.

**Table 1:** Comparative analysis of regional economic prediction model based parameters for APDREO dataset.

APDREO dataset			
Parameters	FWA-SVR	LSTM	BVNN_BP
GDP per capita prediction	88	90	92
Prediction accuracy	92	95	97
GDP per primary growth	90	93	95
GDP per secondary growth	89	92	96
Convergence rate	91	93	97

**Table 2:** Comparative analysis of regional economic prediction model based parameters for WEO dataset.

WEO dataset			
Parameters	FWA-SVR	LSTM	BVNN_BP
GDP per capita prediction	94	95	97
Prediction accuracy	96	97	98
GDP per primary growth	85	87	92
GDP per secondary growth	89	93	95
Convergence rate	85	88	91

**Table 3:** Comparative analysis of regional economic prediction model based parameters for AFRREO dataset.

AFRREO dataset			
Parameters	FWA-SVR	LSTM	BVNN_BP
GDP per capita prediction	79	82	86
Prediction accuracy	92	95	98
GDP per primary growth	81	83	87
GDP per secondary growth	83	86	91
Convergence rate	85	89	93

## Data Availability

The data that support the findings of this study are available from the corresponding author upon reasonable request.

## References

[1] Tuo S., Chen T., He H. (2021). A regional industrial economic forecasting model based on a deep convolutional neural network and big data. *Sustainability*.

[2] Dai Y., Su L. (2021). Economic structure analysis based on neural network and bionic algorithm. *Complexity Problems Handled by Advanced Computer Simulation Technology in Smart Cities*.

[3] Gu H. B., Zhang M. (2017). Index analysis of regional economic integration based on internal forces and external forces: a case study of delta urban agglomeration near the Yangtze River. *Journal of Renmin University of China*.

[4] Garg S. (2019). *A Study on the Structure of Neural Networks and the Mathematics behind Backpropagation*.

[5] Platt D. (2021). Bayesian estimation of economic simulation models using neural networks. *Computational Economics*.

[6] Chc A., Fs A., Fjh B., Wu L. (2020). A probability density function generator based on neural networks. *Physica A: Statistical Mechanics and Its Applications*.

[7] Yeh J.-Y., Chen C.-H. (2020). A machine learning approach to predict the success of crowdfunding fintech project. *Journal of Enterprise Information Management*.

[8] Lv Z., Li Y., Feng H., Lv H. (2021). Deep learning for security in digital twins of cooperative intelligent transportation systems. *IEEE Transactions on Intelligent Transportation Systems*.

[9] Enright C. G., Madden M. G., Madden N. (2013). Bayesian networks for mathematical models: techniques for automatic construction and efficient inference. *International Journal of Approximate Reasoning*.

[10] Tuo S., He H. (2021). A study of multiregional economic correlation analysis based on big data-taking the regional economy of cities in shaanxi Province, China, as an example. *Sustainability*.

[11] Khatwani G., Srivastava P. R. (2018). Impact of information technology on information search channel selection for consumers. *Journal of Organizational and End User Computing*.

[12] Grubljesic T., Coelho P. S., Jaklic J. (2019). The shift to socio-organizational drivers of business intelligence and analytics acceptance. *Journal of Organizational and End User Computing*.

[13] Wu L., Zhang Q., Chen C.-H., Guo K., Wang D. (2020). Deep learning techniques for community detection in social networks. *IEEE Access*.

[14] Yang T., Pan H., Zhang X., Greenlee A., Deal B. (2021). How neighborhood conditions and policy incentives affect relocation outcomes of households from low-income neighborhoods—evidence from intra-city movement trajectories. *Cities*.

[15] Kong L., Liu Z., Wu J. (2020). A systematic review of big data-based urban sustainability research: state-of-the-science and future directions. *Journal of Cleaner Production*.

[16] Allam Z., Dhunny Z. A. (2019). On big data, artificial intelligence and smart cities. *Cities*.

[17] Tang Z., Wang M., Wei D., Liu Z. (2021). Seasonally-adjusted FWA-SVR model and its application in tourism economic forecast. *Journal of Systems Science and Mathematical Sciences*.

[18] Ji Y. (2021). Big data-based mixed frequency macroeconomic prediction and monitoring index construction. *Statistics & Decisions*.

[19] Xia M., Jiang L. (2021). China’s consumer confidence index forecast based on deep network CNN-LSTM model. *Statistics & Decisions*.

[20] Rakpho P., Yamaka W. (2021). The forecasting power of economic policy uncertainty for energy demand and supply. *Energy Reports*.

[21] Lyu Y., Nie J., Yang S.-K. X. (2021). Forecasting US economic growth in downturns using cross-country data. *Economics Letters*.

[22] Claveria O., Monte E., Torra S. (2020). Economic forecasting with evolved confidence indicators. *Economic Modelling*.

[23] Tang X., Li Z., Hu X., Xu Z., Peng L. (2021). Self-correcting error-based prediction model for the COVID-19 pandemic and analysis of economic impacts. *Sustainable Cities and Society*.

[24] Kim S. Y., Upneja A. (2021). Majority voting ensemble with a decision trees for business failure prediction during economic downturns. *Journal of Innovation & Knowledge*.

[25] Huang Y., Wang H., Liu H., Liu S. (2019). Elman neural network optimized by firefly algorithm for forecasting China’s carbon dioxide emissions. *Systems Science & Control Engineering*.

[26] Di W., Wang M., Sun X., Han G., Xing H. (2020). Identification of bolt anchorage defects based on Elman neural network optimised by improved chicken swarm optimisation algorithm. *Insight - Non-Destructive Testing and Condition Monitoring*.

[27] Yang J., Wen J., Jiang B., Wang H. (2020). Blockchain-based sharing and tamper-proof framework of big data networking. *IEEE Network*.

[28] Cai J., Yang L., Zeng C., Chen Y. (2021). Integrated approach for ball mill load forecasting based on improved EWT, refined composite multi-scale dispersion entropy and fireworks algorithm optimized SVM. *Advances in Mechanical Engineering*.

[29] Behera R. K., Jena M., Rath S. K., Misra S. (2021). Co-LSTM: convolutional LSTM model for sentiment analysis in social big data. *Information Processing & Management*.

[30] Kumar A., Shankar R., Aljohani N. R. (2020). A big data driven framework for demand-driven forecasting with effects of marketing-mix variables. *Industrial Marketing Management*.

[31] Raut R. D., Mangla S. K., Narwane V. S., Gardas B. B., Priyadarshinee P., Narkhede B. E. (2019). Linking big data analytics and operational sustainability practices for sustainable business management. *Journal of Cleaner Production*.

[32] Ghayekhloo M., Azimi R., Ghofrani M., Menhaj M. B., Shekari E. (2019). A combination approach based on a novel data clustering method and Bayesian recurrent neural network for day-ahead price forecasting of electricity markets. *Electric Power Systems Research*.

